# Case Report: Strumal carcinoid tumor in the ovary: report of a rare occurrence with a review of literature

**DOI:** 10.3389/fmed.2025.1685641

**Published:** 2025-10-30

**Authors:** Wei Zhu, Yuance Xu, Qin Yao

**Affiliations:** ^1^Department of Obstetrics and Gynecology, The Affiliated Hospital of Qingdao University, Qingdao, China; ^2^Department of Obstetrics and Gynecology, Jilin Women and Children Health Hospital, Changchun, China

**Keywords:** strumal carcinoid tumor of the ovary, ovarian carcinoid tumor, diagnosis, treatment, tumor markers

## Abstract

**Background:**

Primary ovarian carcinoid is a rare type of tumor that accounts for 0.5 to 1.7% of all carcinoid tumors and 1% of all ovarian cancers. These tumors can be classified into four types based on their histopathological features: island-like, trabecular, oral, and mucinous. Island-like carcinomas are more common in Western countries, while chain and trabecular carcinomas are more common in Asian countries. Ovarian strumal carcinoid, a specific type of ovarian tumor that often contains benign thyroid tissue, is a highly differentiated teratoma characterized by the co-existence of thyroid follicular and carcinoid tissues, along with neuroendocrine functions. Preoperative diagnosis is often challenging due to the occult nature and radiographic diversity of the tumor.

**Case report:**

This case report details the diagnosis and treatment of pelvic masses in a 42-year-old patient. The patient had a 3-year clinical history. Ultrasound examination revealed an uneven echo mass measuring 5.1 × 4.0 × 4.3 cm in the right adnexal area with internal striped blood flow signals. However, the levels of CA-125, alpha-fetoprotein (AFP), free T4, thyroid stimulating hormone (TSH), and other diagnostic indicators were all within the normal range. The patient underwent laparoscopic resection of the right adnexa, and the histopathological findings confirmed the presence of an ovarian strumal carcinoid. The patient had a history of persistent constipation and, at the time of the study, reported right upper abdominal distension without dizziness, weakness, lower abdominal distension discomfort, and/or frequent urination. There had been no significant changes in her weight. At the 1-year postoperative follow-up, the patient showed no evidence of recurrence.

**Discussion:**

Through a literature review, we discuss the clinical, imaging, and pathological features of this disease, as well as the outcome of surgical treatment. This report highlights the importance of careful evaluation and timely surgical intervention of pelvic masses. It also emphasizes that rare conditions such as ovarian strumal carcinoid should be taken into account when evaluating atypical ovarian tumors.

## 1 Introduction

Ovarian strumal carcinoid, a rare subtype of ovarian teratoma, is characterized by the presence of mature thyroid tissue within the tumor, which exhibits dysplastic characteristics in the ovary (1). Although the majority of ovarian strumal carcinoids maintain benign biological behavior, there are some cases where they may progress to malignancy, including papillary thyroid carcinoma and insular carcinoid (2). Epidemiologically, ovarian strumal carcinoids account for 0.2 to 1.5% of all ovarian tumors and 5 to 10% of all teratomas. The tumor is more common in women between the ages of 40 and 60 years, although it is also found in younger or postmenopausal women (3). In terms of clinical presentation, ovarian strumal carcinoid presents a variety of clinical characteristics and lacks specific features; the most common manifestation is a pelvic mass, which may be accompanied by abdominal distension or discomfort. If the tumor is large or located in a specific area, patients may experience abdominal pain or pressure, and very few patients present with ascites and elevated CA125 levels (4, 5). In addition, if the tumor shows significant functionality, the patient may experience symptoms of hyperthyroidism, such as palpitations, excessive sweating, or weight loss. However, it is essential to note that the majority of patients have negative laboratory test results for thyroid dysfunction (6).

## 2 Case introduction

**Figure 1:**
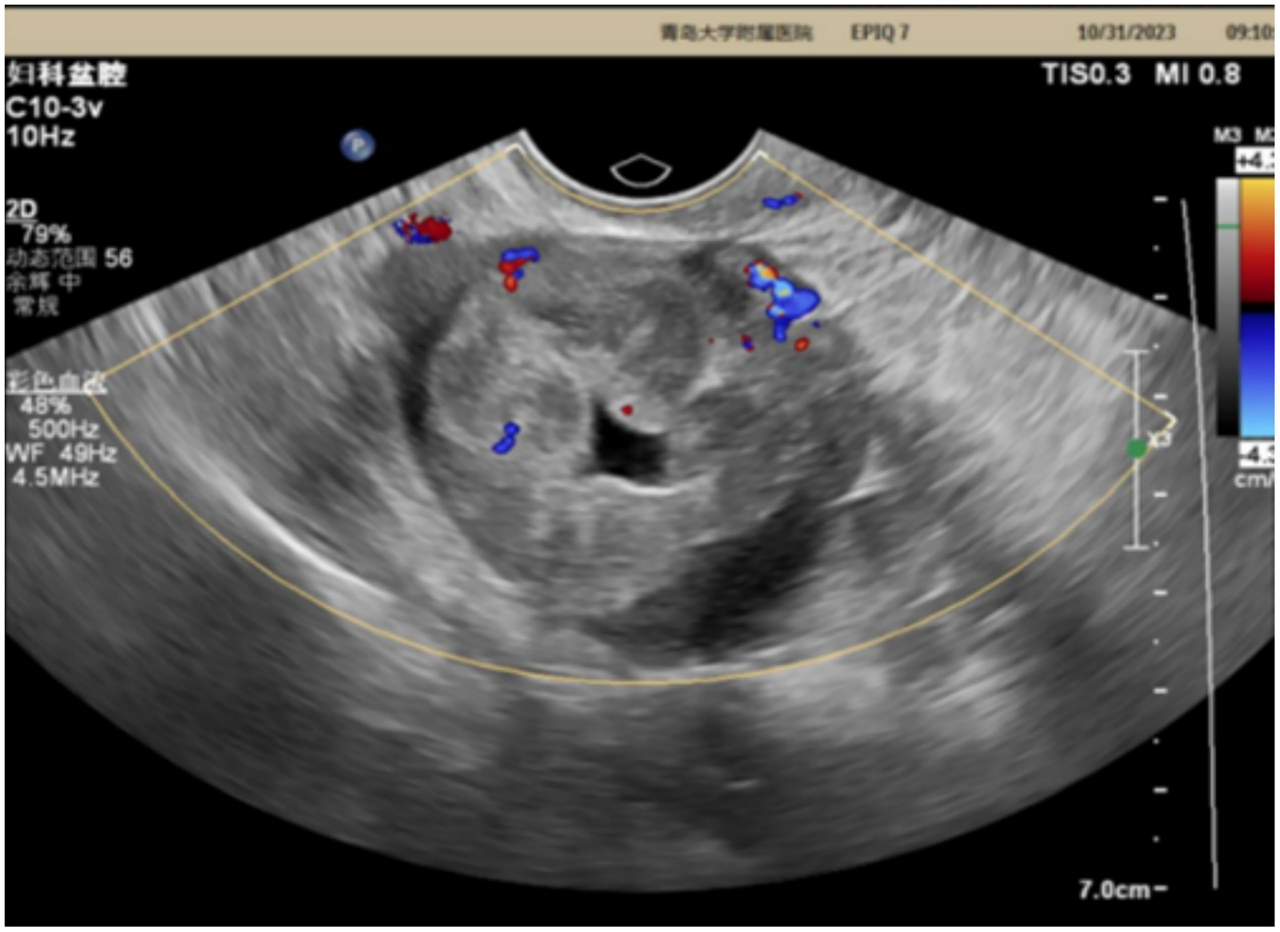
Ultrasonic image.

**Figure 2:**
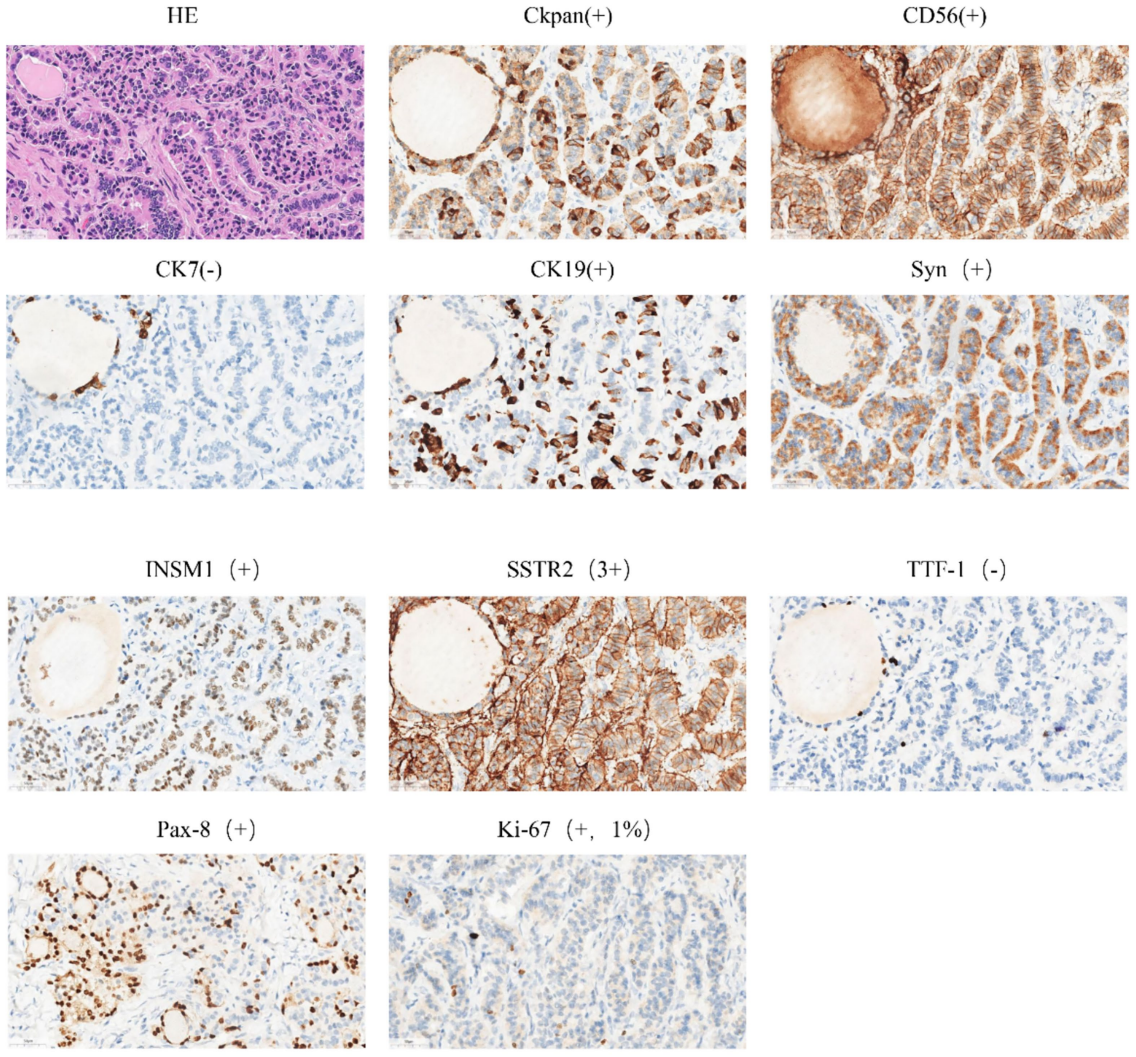
Immumohistochemical staining.

The patient is a 42-year-old married woman (G1P1) with a regular menstrual cycle of 5–6/25 days. She was admitted to the gynecology department of The Affiliated Hospital Of Qingdao University on 1 November 2023, due to her primary complaint of a “pelvic tumor found during a physical examination 3 years ago.” During this examination, an ultrasound revealed a right ovarian cyst measuring 3 cm in diameter. After admission, a follow-up ultrasound showed the following: an uneven echo mass in the right uterine adnexa, measuring 5.1 × 4.0 × 4.3 cm, an irregular echoless area in the center, and a striped blood flow signal in the center (Figure 1). Upon gynecological examination, a 5 cm palpable solid mass with good motion and no tenderness was identified in the right adnexal area, and no palpable abnormality was found in the left adnexal area. Sex hormone FSH:4.57 IU/L; LH:2.85 IU/L; PRL:311.54 mIU/L; E2:290.08 pmol/L; and AMH 1.80 ng/mL; thyroid hormones: FT3:4.00 pmol/L; FT4:14.10 pmol/L; and TSH:2.530 mIU/L; and tumor markers: CA125: 16.18 U/mL; CA199: 23.65 U/mL; CA153: 6.07 U/mL; AFP: 2.01 ng/mL; CEA: 3.29 ng/mL; and HE4: 54.94 pmol/L. The preoperative diagnosis indicated a right ovarian cyst with the potential for malignancy. Later, an elective laparoscopic oophorectomy of the right ovary was performed. During the procedure, solid masses of approximately 5 cm in diameter were found in the right ovary, which were hard in texture. No obvious abnormalities were found in the appearance of both fallopian tubes and the left ovary, and no obvious ascites was observed in the abdominal cavity. No enlarged lymph nodes were palpated in the pelvic cavity. The right pelvic funnel ligament, the root of the right fallopian tube, and the proper ligament of the right ovary were disconnected, and the right adnexa along the right mesosalpinx and the posterior leaf of the right broad ligament were removed. The right adnexa was placed into a specimen bag and removed through the puncture site. The tumor did not rupture during surgery. The surgery lasted 1 h, with an estimated blood loss of approximately 10 mL, and the patient had no significant perioperative complications. Considering that this was a borderline malignancy, the ovarian surface remained intact and no tumor tissue was observed; hence, peritoneal washings were not retained. Gross appearance: the right fallopian tube was 5.5 cm long with a maximum diameter of 0.8 cm, and the fimbrial end was visible. The right ovary was cystic, measuring 5 × 4 × 2.7 cm, with a gray-yellow solid cut surface. Observed under the microscope: Trabecular and insular carcinoid component and colloid-filled thyroid follicles without cytological atypia. No capsular invasion and necrosis, and mitotic count < 1/10 high-power field (HPF). Intraoperative freezing: The right ovary was considered carcinoid, and a small amount of thyroid follicular epithelium was found, which was initially misinterpreted as carcinoid tissue within the ovary. Postoperative pathology: (right ovary) consistent with struma carcinoid. Immunohistochemistry (IHC): CKpan(+), CD56(+), CK7(−), CK19(+), Syn(+), insulinoma-associated protein 1 (INSM1; +), somatostatin receptor type 2 (SSTR2; 3+), TTF-1(−), Pax-8(+), and Ki-67(1%; +) (Figure 2). IHC was mainly performed on the carcinoid areas, and the differentiated thyroid regions were not subjected to detailed IHC, or the proportion of differentiated thyroid tissue sampled was small. We believe this may explain why TTF-1 was negative and synaptophysin (Syn) was positive on slide review. The diagnosis was confirmed by two gynecological pathologists and discussed within the department. External consultation was not required, but is noted as advisable for rare mixed lesions. Postoperative diagnosis: right ovarian strumal carcinoid (stage IA). Patients were closely followed at 1, 3, 6, and 12 months postsurgery. Ultrasonography depicted that the size of both ovaries was normal, and no abnormal echo region was found. No abnormalities were found in the tumor index and thyroid and sex hormones. The patient has not yet reached 24 months postsurgery, which is one limitation of this case; follow-up should continue.

Syn, CD56, and INSM1: confirmed neuroendocrine differentiation. PAX8 and CK19: support thyroid follicular origin. TTF-1 negativity: recognized but not universal in struma; absence of thyroglobulin staining limits absolute proof of thyroid origin—noted as limitation. SSTR2 3+: may allow Ga-68 DOTATATE imaging or somatostatin analogue therapy if clinically indicated.

## 3 Literature discussion and review

The PubMed database was used to conduct a literature review of English original articles, review articles, and case reports on ovarian thyroid carcinoid tumors. The search terms were ovarian thyroid carcinoid, thyroid carcinoid case report, and thyroid carcinoid tumors of the ovaries. Inclusion: case reports, case series, reviews with histologically confirmed ovarian strumal carcinoid. Exclusion: non-English, abstracts only, duplicate publications, and non-ovarian sites. We searched for studies on ovarian carcinoid tumors from 2020 to 2025 (date of last search: May 31, 2025) and collected a total of 42 related articles. After applying exclusion criteria, we retrospectively analyzed a total of 10 studies (Table 1).

**Table 1:** Collected cases and patient characteristics.

No.	Authors (year)	Patients (*N*)	Clinical manifestations	Mean size	Tumor marker	Classification	IHC	Concomitant Tumors
1	Turla et al. (2022) (2)	1	Abdominal pain, constipation	4.1	Normal	Strumal	CgA+, CK7+, CD56+, Ki-67:2%, TTF-1	Absent
2	Fathi Mraihi (2024) (24)	1	Abdominal distension	15.8	Normal	Trabecular	CgA+, CD56+, TTF-1	Absent
3	Li-Ping Shen (2022) (25)	1	Abdominal mass	6	Normal	Strumal	CK7+, Syn++, CgA++, Ki-67:5%	Absent
4	Mohammed (2021) (26)	1	Abdominal pain, anorexia frequent urination	24	CA125:105 IU/mL, CEA:6.4 ng/ML	Insular, trabecular	Syn, CgA+	Mature cystic teratoma
5	Turla et al. (2022) (2)	1	Abdominal pain, constipation	4	Normal	Absent	Syn, CgA+, CD56+, CK7+, Ki-67: 2%	Absent
6	Yuan (2022) (27)	1	Absent	9	Absent	Strumal	CD56+, NSE+, PSAP+, CDX-2 +, AE1/AE3 +	Mature cystic teratoma
7	Baruah et al. (2022) (15)	1	Abdominal distension	15	CA125:944 IU/mL	Strumal	Syn+, CD56+, PAX8+, Ki-67: 3%	Mature cystic teratoma, Appendix carcinoid
8	Cagino (2022) (28)	1	Abdominal pain, colporrhagia	10	absent	Insular	NCE+, Syn+, CgA +	Mature cystic teratoma
9	Mohammed (2021) (26)	1	Abdominal pain	24	CA12:105 U/mL; CEA: 6.4 ng/ml	Insular and trabecular	TTF-1	Absent
10	Liu (2025) (29)	1	Abdominal pain	9.5	Normal	Insular, trabecular	TTF-1, CDX-2 +, PAX-8+, CD56+	Absent

### 3.1 Clinical features

Strumal ovarian carcinoid is a rare ovarian tumor belonging to a type of ovarian teratoma, which is composed of mature thyroid tissue. These tumors are more common in middle-aged women, especially in later reproductive years (7). Although most ovarian strumal carcinoids remain benign, they may also contain malignant potential, requiring careful clinical evaluation and appropriate treatment strategies. In the early stages, many patients may not show significant symptoms, often resulting in the incidental discovery of the tumors during routine physical examinations or other surgeries. As the tumor grows in size, patients may gradually experience non-specific symptoms such as pelvic discomfort, bloating, or abdominal pain. These masses may be unilateral or bilateral, vary in size and shape, and are usually relatively mobile and not accompanied by tenderness (4). In the latest article, Turla A et al. collected data on 117 patients, studied the latest clinical features, and found that 37% of patients presented with abdominal bulge, 49% presented with pain caused by the enlargement of abdominal tumor masses, and 37% presented with constipation (only 9 patients underwent peptide YY [PYY] analysis). Results beyond the physiological range, many authors suggest that the release of PYY peptides is a possible cause of constipation symptoms in patients with struma carcinoid cancer (8). Serum PYY, serotonin, and 24-h urinary 5-hydroxy indoleacetic acid (5-HIAA) were not measured in this case because carcinoid syndrome was not suspected clinically; this represents a retrospective limitation. Future cases of chronic constipation in non-malignant ovarian tumors should include peptide profiling to enhance functional relevance. In addition, we have seen different clinical features in the literature, such as abnormal uterine bleeding, frequent urination, and lower limb edema (9). In cases where the tumor shows significant functionality, patients may experience symptoms associated with hyperthyroidism, including palpitations, excessive sweating, and weight loss. Despite this, many patients continue to exhibit thyroid function test results in the normal range (6). Some strumal carcinoid patients also exhibit typical signs and symptoms of carcinoid syndrome, such as intermittent flushing, abdominal cramps, diarrhea, carcinoid heart disease, etc., which are mediated by bioactive substances produced by carcinoid cancer cells. In addition, tumor markers such as CA-125 may be elevated in some cases, but their specificity is usually low (5).

### 3.2 Imaging feature

Ovarian strumal carcinoid exhibits unique features in imaging examinations such as ultrasound, computed tomography (CT), and Magnetic Resonance Imaging (MRI), which are essential for diagnosis and differential diagnosis. On ultrasound, ovarian strumal carcinoid often presents as a mixed echogenic mass in the pelvic cavity. Its internal structure is complex and varied and may contain both cystic and solid components. This pattern of echoes, particularly uneven internal echoes and cellular or cystic areas similar to thyroid tissue, provides important clues for diagnosis (10). CT scans further reveal the size and shape of the tumor and can detect features such as calcification, helping assess the density of the tumor and its relationship to surrounding structures, such as potential invasion of neighboring organs or blood vessels. MRI, which has a high contrast for soft tissues, can provide more detailed information about the internal structure of the tumor. In the T2-weighted sequence, thyroid tissue components of ovarian strumal carcinoid typically show high signaling, while cystic areas show significantly low signaling, helping distinguish between cystic and solid parts of the tumor. In T1-weighted sequences, thyroid tissue may show equal or slightly higher signals, in contrast to surrounding ovarian tissue. The multiparameter imaging technique of MRI is particularly important in complex cases, especially when evaluating internal bleeding and necrosis or fat composition of the tumor, and helps further distinguish between benign and malignant tumors (11). Dynamic enhancement of color Doppler ultrasound (CDFI) and MRI is an important means to evaluate the blood supply of tumors. Malignant tumors may exhibit rich blood flow signals and rapid enhancement patterns during these examinations. This information is crucial for assessing the biological behavior of the tumor prior to surgery and for developing surgical plans. Although imaging features are of great value in the diagnosis of ovarian strumal carcinoid, the diagnosis still depends on pathological examination. Imaging provides critical information for preoperative evaluation and helps predict the malignant potential of the tumor. However, given the rarity of ovarian strumal carcinoid and the diversity of imaging findings, its features may be similar to other ovarian tumors. Hence, in cases where the imaging features are atypical, a more comprehensive evaluation, including laboratory tests and histological analysis, should be performed to ensure an accurate diagnosis.

### 3.3 Pathological features

Struma ovary carcinoid is a type of ovarian teratoma containing thyroid tissue, and its pathological features play a crucial role in diagnosis and differential diagnosis. Ovarian carcinoid tumors have been divided into four groups based on histopathological patterns: insular, trabecular, oral, and mucinous (12). Among them, a mixture of trabecular and insular types is common. Homogeneous islands of tumor cells are typical of insular carcinoids. Trabecular carcinoids are characterized by tumor cells growing in trabecular cells, with few endocrine manifestations (13). IHC analysis revealed a similar immunophenotype of ovarian strumal carcinoid with normal thyroid tissue, wherein the strong positive reaction of thyroid-specific transcription factor-1 (TTF-1) and PAX8 was a significant feature (14, 15). These transcription factors are essential for the development and differentiation of thyroid tissue, and their presence not only confirms the thyroid origin of tumors but also provides pathologists with key clues for differential diagnosis. Thyroglobulin, another common marker, is also often detected in the follicles of struma carcinoid, further confirming the thyroid tissue characteristics of the tumors. In view of the possible malignant changes in the tumor, such as papillary thyroid cancer (PTC), IHC analysis provides strong evidence for diagnosis by identifying positive responses of HBME-1 and calretinin (16). In addition, the Ki-67 index of malignant ovarian strumal carcinoid is often higher, reflecting the increased proliferative activity of tumor cells (14).

On pathological examination, the positive response of the carcinoid components of ovarian strumal carcinoid to neuroendocrine markers such as synaptophysin and chromogranin A helps the pathologist distinguish between carcinoid and thyroid tissue within the tumor, especially when the two tissues are mixed (17). IHC analysis is particularly important in differential diagnosis, especially in distinguishing between primary ovarian PTC and ovarian metastases of thyroid cancer, which may share similar histological and immunophenotypic characteristics with the former. At this point, a detailed clinical history and radiological evaluation are essential to determine the origin of the tumor. The different expression patterns of IHC markers, such as TTF-1, in ovarian epithelial and thyroid tissue provide a powerful tool for pathologists to distinguish between the two conditions (18).

Notably, thyroid tissue in ovarian strumal carcinoid may exhibit functional changes similar to normal thyroid, such as chronic lymphocytic thyroiditis and papillary hyperplasia, which can be confirmed by IHC analysis for specific inflammatory cell markers such as CD3, CD4, and CD8 (17).

In summary, IHC analysis plays an indispensable role in the diagnosis and differential diagnosis of ovarian strumal carcinoid. By utilizing a range of antibodies that target thyroid-specific and neuroendocrine markers, pathologists are able to accurately identify and classify ovarian strumal carcinoids and their malignant changes, providing valuable information for clinical treatment decisions.

### 3.4 Therapy

The core strategy for the treatment of ovarian strumal carcinoid is surgical resection, which is the most effective treatment modality. The type and scope of surgery should take into account tumor size, location, potential risk of malignancy, and the patient’s reproductive needs. For ovarian strumal carcinoids that are considered benign, conservative surgery, such as unilateral oophorectomy, is usually recommended to maximize the patient’s fertility. The primary goal of the surgery is to completely remove the tumor while preserving as much healthy ovarian tissue as possible (19).

In the presence of larger tumors or signs of malignancy, more extensive surgery may be required, such as total hysterectomy or bilateral salpingo-oophorectomy. If the tumor has invaded adjacent structures, pelvic lymph node dissection or peritonectomy may also be required. In some cases, if preoperative or intraoperative evaluation reveals the malignant potential of the tumor, postoperative adjuvant therapy may be necessary. Adjuvant therapy may include radiotherapy, chemotherapy, or targeted therapy, wherein radiotherapy is primarily used to control local lesions, chemotherapy is used for potentially small metastatic lesions or identified distant metastases, and targeted therapy for thyroid cancers with specific molecular markers, such as papillary thyroid cancer with BRAF mutation (20).

For malignant ovarian strumal carcinoid confirmed by pathological examination after surgery, the treatment plan should be personalized according to the histological classification of the tumor, clinical stage, and the overall health status of the patient. For example, patients with papillary thyroid cancer may be treated with radioactive iodine, while other types of thyroid cancer may require chemotherapy or molecularly targeted therapy. During treatment, the patient’s thyroid function needs to be closely monitored, as the presence of tumors may cause fluctuations in thyroid hormone levels. For patients who have undergone extensive surgery or are at risk for thyroid dysfunction, thyroid hormone replacement therapy may be required to maintain normal physiological function.

### 3.5 Prognosis

Patients with strumal ovarian carcinoid generally enjoy a more optimistic prognosis, especially when the tumor has been completely removed and no malignant transformation has occurred. However, the actual direction of prognosis may be influenced by a number of factors, including tumor size, histological features, the presence or absence of malignant changes, and the timeliness and thoroughness of treatment. In most cases, patients with benign ovarian strumal carcinoid who have undergone surgical removal show a good prognosis, with a low recurrence rate and a high long-term survival rate (21). These patients are usually able to maintain a normal life and fertility without the need for additional adjuvant therapy. Conversely, if the tumor is large or shows functional activity, the patient may need more frequent monitoring to ensure stable thyroid function. For those ovarian strumal carcinoids that have undergone malignant transformation, such as papillary thyroid carcinoma or undifferentiated carcinoma, the prognosis may be relatively poor. This type of tumor may carry a higher risk of recurrence and distant metastasis, especially if the tumor has invaded tissues outside the ovary or there are distant metastases. In these cases, in addition to surgical resection, patients may also need to undergo radiation therapy, chemotherapy, or targeted therapy to reduce the risk of recurrence and improve survival. IHC features, such as Ki-67 index, which is a significant prognostic marker (22). As a marker of cell proliferation, the expression level of Ki-67 in tumor cells is closely related to tumor aggressiveness and prognosis, and tumors with a higher Ki-67 index may indicate a higher risk of recurrence and a poorer prognosis.

The stage of the tumor is also one of the key factors determining its prognosis. Early stage tumors (FIGO stages I-II) usually have a favorable prognosis, while advanced tumors (FIGO stages III-IV) may require more intensive treatment and exhibit a relatively poor prognosis (23). Certain histological types of tumors, such as highly differentiated follicular thyroid cancer, may be associated with a better prognosis. In addition, age of the patient, the overall state of health, and the quality of treatment also significantly impact the prognosis. Young, healthy patients, especially those treated at specialized medical centers, tend to have a better prognosis.

Overall, the prognosis of ovarian strumal carcinoid is influenced by a combination of factors, including the nature of the tumor, the timeliness and thoroughness of treatment, and individual patient differences. For benign tumors, the prognosis is generally positive. For malignant transformed tumors, the prognosis may be more complex, requiring comprehensive treatment and strict monitoring. With the growing understanding of such diseases and advancement in treatment technology, the prognosis of patients is expected to be further enhanced.

## 4 Conclusion

As a rare ovarian tumor containing mature thyroid tissue, the diagnosis and treatment strategy of strumal ovarian carcinoid has special significance in medicine. To ensure the timeliness and clinical relevance of the study, the case data collected in this research spans the period from 2020 to 2025, while acknowledging that a wider timespan would yield additional cases. According to literature review and case reports, the disease is more common in middle-aged women, and the clinical manifestations often lack specificity, with common symptoms such as pelvic masses or discomfort. Although it is difficult to make a definitive diagnosis before surgery, strumal ovarian carcinoid presents the characteristics of PYY peptide release, endocrine dysfunction, steroid hormone secretion, etc. Therefore, for female patients with chronic habitual constipation, abdominal cramps, diarrhea, and menstrual abnormalities, the occurrence of this rare ovarian malignant tumor should be considered. During diagnosis, imaging, especially ultrasound, plays a crucial role in revealing the size, shape, and nature of the tumor. Pathological examination and IHC analysis provide conclusive evidence for diagnosis, in which the tumor’s positive response to thyroid-specific markers, such as TTF-1, PAX8, and thyroglobulin, is key to diagnosis.

In terms of treatment, surgical resection is the treatment of choice for ovarian strumal carcinoid, usually involving tumor removal or unilateral ovariectomy. Fertility-sparing unilateral salpingo-oophorectomy is appropriate in young women with stage IA disease. For tumors that exhibit malignant changes, more extensive surgery may be required, including total hysterectomy and pelvic lymph node dissection, as well as omentectomy. In the case of malignant ovarian strumal carcinoid, the prognosis may be relatively poor and a comprehensive treatment strategy, including postoperative chemotherapy or radiotherapy, is particularly important. In contrast, the prognosis of benign ovarian strumal carcinoid is generally more optimistic, especially when the tumor is completely removed and no signs of malignancy are found. Postoperative management includes thyroid ultrasound imaging and testing of TSH values every 12 months for 5 years. The patient should be referred to endocrinology if nodules or dysfunction develop.

In summary, the clinical management of ovarian strumal carcinoid requires close collaboration between a multidisciplinary team of gynecological oncologists, pathologists, radiologists, and endocrinologists. Through accurate clinical evaluation, careful imaging, pathological diagnosis, and appropriate surgical treatment, most patients are expected to achieve good treatment outcomes and quality of life. For potential malignant changes, timely and accurate diagnosis, along with proper treatment, are key to improving patient prognosis.

## Data Availability

The raw data supporting the conclusions of this article will be made available by the authors, without undue reservation.
